# Liquid Biopsy Profiling with Multiple Tests in Patients with Metastatic Breast Cancer

**DOI:** 10.3390/jmp5020013

**Published:** 2024-05-09

**Authors:** Nikki Higa, Lisa Welter, Liya Xu, Anand Kolatkar, Kelli S. Bramlett, Ole V. Gjoerup, Ryon Graf, Richard S.P. Huang, Rebecca J. Leary, Young Lee, Jeremy G. Perkins, Adam I. Riker, Angad P. Singh, Lorraine Tafra, Carol K. Tweed, Craig D. Shriver, James Hicks, Peter Kuhn

**Affiliations:** 1 Convergent Science Institute in Cancer, Michelson Center for Convergent Bioscience, University of Southern California, Los Angeles, CA 90089, USA; 2 Department of Medicine, Keck School of Medicine, University of Southern California, Los Angeles, CA 90033, USA; 3 Department of Biological Sciences, Dornsife College of Letters, Arts, and Sciences, University of Southern California, Los Angeles, CA 90089, USA; 4 The Vision Center at Children’s Hospital Los Angeles, Los Angeles, CA 90027, USA; 5 USC Roski Eye Institute, Keck School of Medicine, University of Southern California, Los Angeles, CA 90033, USA; 6 Thermo Fisher Scientific, Austin, TX 78744, USA; 7 Foundation Medicine, Inc., Cambridge, MA 02141, USA; 8 Novartis Biomedical Research, Cambridge, MA 02139, USA; 9 Luminis Health, Anne Arundel Medical Center, DeCesaris Cancer Institute, Annapolis, MD 21401, USA; 10 Murtha Cancer Center Research Program, Department of Surgery, Uniformed Services University of the Health Sciences, Bethesda, MD 20889, USA; 11 Walter Reed National Military Medical Center, Bethesda, MD 20889, USA; 12 Precision Healthcare Specialists, Naples, FL 34102, USA; 13 Breast Care Architects, LLC., Crownsville, MD 21032, USA; 14 Maryland Oncology Hematology, Annapolis, MD 21401, USA; 15 Institute of Urology, Catherine & Joseph Aresty Department of Urology, Keck School of Medicine, University of Southern California, Los Angeles, CA 90033, USA; 16 Norris Comprehensive Cancer Center, Keck School of Medicine, University of Southern California, Los Angeles, CA 90033, USA; 17 Department of Biomedical Engineering, Viterbi School of Engineering, University of Southern California, Los Angeles, CA 90089, USA; 18 Department of Aerospace and Mechanical Engineering, Viterbi School of Engineering, University of Southern California, Los Angeles, CA 90089, USA

**Keywords:** liquid biopsy, breast cancer, cell-free DNA, circulating tumor cells

## Abstract

The chief goal of the Blood Profiling Atlas in Cancer (BloodPAC) consortium is to promote collaborative efforts that support the development and implementation of liquid biopsy tests. Here, we report the results of a pilot study conducted by three BloodPAC members that aimed to demonstrate a multisite liquid biopsy testing framework using longitudinal blood specimens from 38 patients with metastatic breast cancer. Three laboratories receiving identical samples from two clinical sites each applied a different targeted sequencing platform to analyze mutations in cell-free DNA (cfDNA). The resulting mutational profiles reflected common breast cancer alterations, including clinically actionable mutations for 40% of hormone- receptor-positive patients. In 12 genes with shared target regions across sequencing panels, perfect inter-assay concordance was also observed for mutations detected above the lowest common assay limit of detection. Whole-genome copy number profiling of cfDNA and circulating tumor cells (CTCs) further revealed marked heterogeneity in copy number alterations and cfDNA tumor fractions across patients. Additionally, comparison of tumor fraction and CTC abundance demonstrated the complementary nature of cfDNA and CTC analyses. Overall, the framework described in this study may serve as a resource for future trials aiming to identify multimodal liquid biopsy biomarkers to guide clinical care.

## 1. Introduction

The ability of cancer cells to seed distant sites and evolve under treatment pressure is widely regarded as a key challenge to effective disease management in breast cancer [1,2]. Understanding the biological and therapeutic consequences of this spatiotemporal heterogeneity is important to developing an appropriate treatment strategy; however, tissue biopsies provide only a limited view of global tumor characteristics and require invasive procedures [3,4]. In contrast, liquid biopsies (LBxs) are minimally invasive and provide the opportunity for repeatable testing as well as the potential to capture tumor-associated analytes from multiple lesions [5,6]. As such, LBxs are an attractive complement to guide precision medicine approaches and are the subject of many active pre-clinical and clinical investigations.

Multiple LBx analytes such as circulating cell-free DNA (cfDNA), circulating tumor cells (CTCs), and extracellular vesicles (EVs) have displayed clinical relevance in breast cancer. For example, sequencing of cfDNA has produced mutational profiles reflective of primary and metastatic lesions [5,7,8] and is being applied to detect *ESR1* and *PIK3CA* mutations corresponding to FDA-approved targeted therapies [9,10]. The fraction of cfDNA that is tumor-derived (ctDNA) has also been associated with tumor burden and explored as both a prognostic and predictive biomarker [11–13]. Similarly, CTC abundance of 5 CTCs per 7.5 mL of blood is associated with both decreased overall and progression free survival in metastatic breast cancer (MBC) [14]. Molecular profiling of CTCs has further been applied to assess therapeutically relevant disease features such as endocrine resistance and an epithelial to mesenchymal transition state [15,16]. Finally, approaches incorporating multiple analytes are also being tested to overcome limited quantities of tumor-associated circulating species and maximize the information obtained from a LBx sample [17,18].

With the growing number of LBx platforms in development, generating robust analytical and clinical evidence to demonstrate their validity and utility will be critical for clinical adoption [19]. However, despite considerable research efforts, only one CTC enumeration test and three cfDNA-based tests for *ESR1* or *PIK3CA* mutations have obtained FDA-approval for indications in MBC [20–22]. A key challenge to bringing more LBx tests to the clinic has been the lack of standardized guidance to inform appropriate study designs to support clinical utility, reproducibility, regulatory approval, and reimbursement [23,24]. The increasing complexity of emerging applications, such as characterizing cfDNA fragment features and CTC phenotypes, could further accentuate the issue.

In response to challenges like this, the Blood Profiling Atlas in Cancer (BloodPAC) consortium was established in 2016, bringing together stakeholders across academia, private foundations, industry, and the government with the goal of aiding the development and approval of LBx assays to improve patient outcomes [23]. One of the early outcomes of the consortium was the BloodPAC Data Commons, created as a standardized and secure repository for LBx data that could provide evidence to bring more LBx tests into routine clinical practice [25]. To support this effort, BloodPAC members initiated this pilot study to collect MBC patient samples and perform a multiplatform blood profiling analysis, with the aim to accumulate real-world data and experience to inform further development of the Data Commons and guide future clinical development of LBx assays to impact patient care. Here, we describe a framework for multicenter LBx testing and report genomic findings from analyses of cfDNA and CTCs in longitudinal blood samples from 38 patients with MBC.

## 2. Materials and Methods

### 2.1. Patient Population

From 2018 to 2020, the BloodPAC-007 study enrolled patients with breast cancer at the Walter Reed National Military Medical Center Murtha Cancer Center (WRNMMC MCC) and Anne Arundel Medical Center (AAMC). Approval for this study was granted by both the WRNMMC IRB (WRNMMC-2018-0130) and AAMC IRB (AAMC-1109045), and written informed consent was obtained from all study participants. Eligibility criteria included individuals aged 18 or older with a diagnosis of breast cancer with metastatic disease who were starting a new line of therapy at enrollment. Both chemotherapy- and hormone-therapy-treated patients were eligible.

### 2.2. Sample Collection

Blood collection was scheduled for cycle 1, day 1 and cycle 2, day 1 of a new therapy. Additional draws could occur if there was a disease progression or change in treatment for any reason. These were also collected prior to the first dose in cycles 1 and 2 of each subsequent therapy, for up to 48 months from study enrollment. At each study visit, three peripheral blood samples were collected and distributed to laboratories at Foundation Medicine (FMI), Novartis Institutes for Biomedical Research (NIBR), and the University of Southern California (USC). Whole blood collected in 10 mL Streck tubes was shipped at room temperature to FMI and USC. For NIBR, whole blood in a 10-mL EDTA tube was processed within three hours of blood collection to isolate plasma via centrifugation at room temperature at 1600 × *g* for 10 min followed by 3000 × *g* for 10 min. The plasma was then frozen at −80 °C in 2 mL cryogenic vials and shipped on dry ice.

### 2.3. FMI FoundationACT Assay

Sample processing, library preparation, and sequencing analysis were performed in accordance with the FoundationACT assay (previous generation of FMI’s current onmarket liquid CGP assay, FoundationOneLiquid CDx [26]) by Foundation Medicine’s CAP, CLIA laboratory [27]. A minimum of 20 ng of extracted cfDNA was used for library construction and adaptor-ligated libraries were created using custom molecular and sample barcodes. Solution hybrid capture was performed using a set of biotinylated oligonucleotide baits designed against 62 genes. Captured libraries were purified and normalized to 1.05 nmol/L prior to being pooled and sequenced using the Illumina HiSeq platform (Illumina, San Diego, CA, USA) with 150 bp paired-end reads to a depth of >5000 × unique coverage. Sequencing reads were processed using a previously described computational pipeline [27], utilizing custom methods to correct errors <0.05%, and designed to detect base substitutions (down to 0.1% allele frequencies), short insertions and deletions (down to 1%), rearrangements/fusions (down to 1%), and copy number amplifications (>20%). Filtered variants were annotated as known or likely functional driver alterations based on presence in the Catalogue Of Somatic Mutations In Cancer (COSMIC) [28] or general knowledge in the scientific literature, while all other uncharacterized alterations were classified as variants of unknown significance.

### 2.4. NIBR PanCancer ctDNA Assay

Plasma processing, library preparation, and sequencing analysis for the cfDNA Pan-Cancer assay were performed as previously reported [29]. cfDNA was extracted from approximately 4 mL of plasma (QIAamp Circulating Nucleic Acid Kit, QIAGEN, Germantown, MD, USA) per the manufacturer’s instructions. Sequencing libraries were constructed (TruSeq Nano Library Preparation Kit, Illumina, San Diego, CA, USA) and enriched using a custom designed set of biotinylated oligonucleotide baits designed for the exons and selected introns of 567 genes (2.9 Mb). Capture libraries were normalized, pooled, and sequenced on the Illumina HiSeq platform (Illumina, San Diego, CA, USA) to a target depth of >1000× unique coverage. The sequencing data were aligned to the hg38 reference human genome and variant calling was performed using MuTect v1.1.7 [30] for single nucleotide variants (SNVs), Pindel v1.0 [31] for short insertion/deletion (indel) events, and PureCN v1.16.0 [32] for copy number alterations (CNAs). SNVs and indels were compared to reference databases to remove germline events and sequencing artifacts and then annotated using dbSNP v146 [33], COSMIC v70 [28], and the SnpEff tool v4.3c [34].

### 2.5. USC High-Definition Single Cell Assay (HDSCA) and Thermo Fisher Oncomine Breast cfDNA Assay v2

Blood processing, cfDNA extraction, and CTC detection were carried out using the previously published HDSCA workflow [6,35]. In brief, blood tubes were centrifuged to separate the plasma and cellular fractions. The plasma was stored for cfDNA extraction using the QIAamp Circulating Nucleic Acid Kit (QIAGEN, Germantown, MD, USA). The cellular fraction underwent erythrocyte lysis to obtain nucleated cells, which were plated onto glass slides. Slides were stained and imaged using a four-channel immunofluorescence assay consisting of DAPI, pan-cytokeratin, vimentin, and CD45/CD31. The immunofluorescence images were segmented and clustered to identify rare cell candidates for review. CTCs were manually enumerated by a trained analyst and defined as nucleated, cytokeratin positive cells. For patient 12, CTCs that were also positive in the CD45/CD31 channel were included in the total CTC count based on the presence of clonal alterations in these cells.

Copy number alteration (CNA) profiling was performed using a low-pass whole-genome sequencing (lpWGS) method [6,36], with 5 ng of extracted cfDNA and 50 ng of single cell whole-genome amplification product used as the input for library preparation for cfDNA and single cells, respectively. Libraries were sequenced on an Illumina instrument (NextSeq 500 or HiSeq platform, Illumina, San Diego, CA, USA) using single-end 50 bp or paired-end 150 bp reads to achieve approximately 0.04× coverage. Sequencing reads were aligned to the hg19 reference genome, PCR duplicates were removed, binned read counts were normalized for GC-content, and bin counts across the genome were segmented and represented as ratios to the genome-wide mean. The ichorCNA package [7] was used to estimate the fraction of ctDNA based on the whole-genome copy number profiles. This tool has a reported 91% sensitivity and 100% specificity to detect ctDNA at the tumor fraction threshold of 0.1.

For SNV detection, targeted sequencing libraries were prepared with 20 ng of extracted cfDNA as the input and using the Oncomine Breast cfDNA Assay v2 (Thermo Fisher Scientific, Waltham, MA, USA) according to the manufacturer’s protocol. Due to extracting only 1 mL of plasma at a time, we were not able to obtain sufficient cfDNA from certain samples. Libraries were quantified using the Qubit High-Sensitivity dsDNA Assay (Thermo Fisher Scientific, Waltham, MA, USA) and Ion Library TaqMan Quantitation Kit (Thermo Fisher Scientific, Waltham, MA, USA), and library quality was assessed using the Agilent 2100 Bioanalyzer with High-Sensitivity DNA assay (Agilent Technologies, Santa Clara, CA, USA). Libraries were diluted to the recommended 100 pM, pooled for templating using the Ion 540 kit and Ion Chef instrument (Thermo Fisher Scientific, Waltham, MA, USA), and sequenced on the Ion S5^™^ system (Thermo Fisher Scientific, Waltham, MA, USA). Sequencing data were analyzed using the Torrent Suite Software v5.6.0 and Ion Reporter v5.6. The Oncomine Breast Liquid Biopsy w1.3 DNA Single Sample workflow was used with default parameters for variant calling.

### 2.6. Data Analysis

Variant calls provided by each laboratory were aggregated and matched by sample identifier. Cancer-related genes were obtained from COSMIC [28]. Clinically actionable variants were defined as those considered FDA Level 2: Cancer Mutations with Evidence of Clinical Significance and were obtained from OncoKB v4.2 [37] (Supplementary Table S1). Pearson correlation was used to compare variant allele frequencies (VAFs) reported for pairwise concordant variants. Plots were generated in R v4.1.2 using the ggplot2 v3.3.6 [38] and ComplexHeatmap v2.10.0 [39,40] packages.

## 3. Results

### 3.1. Study Workflow

**Figure 1.**
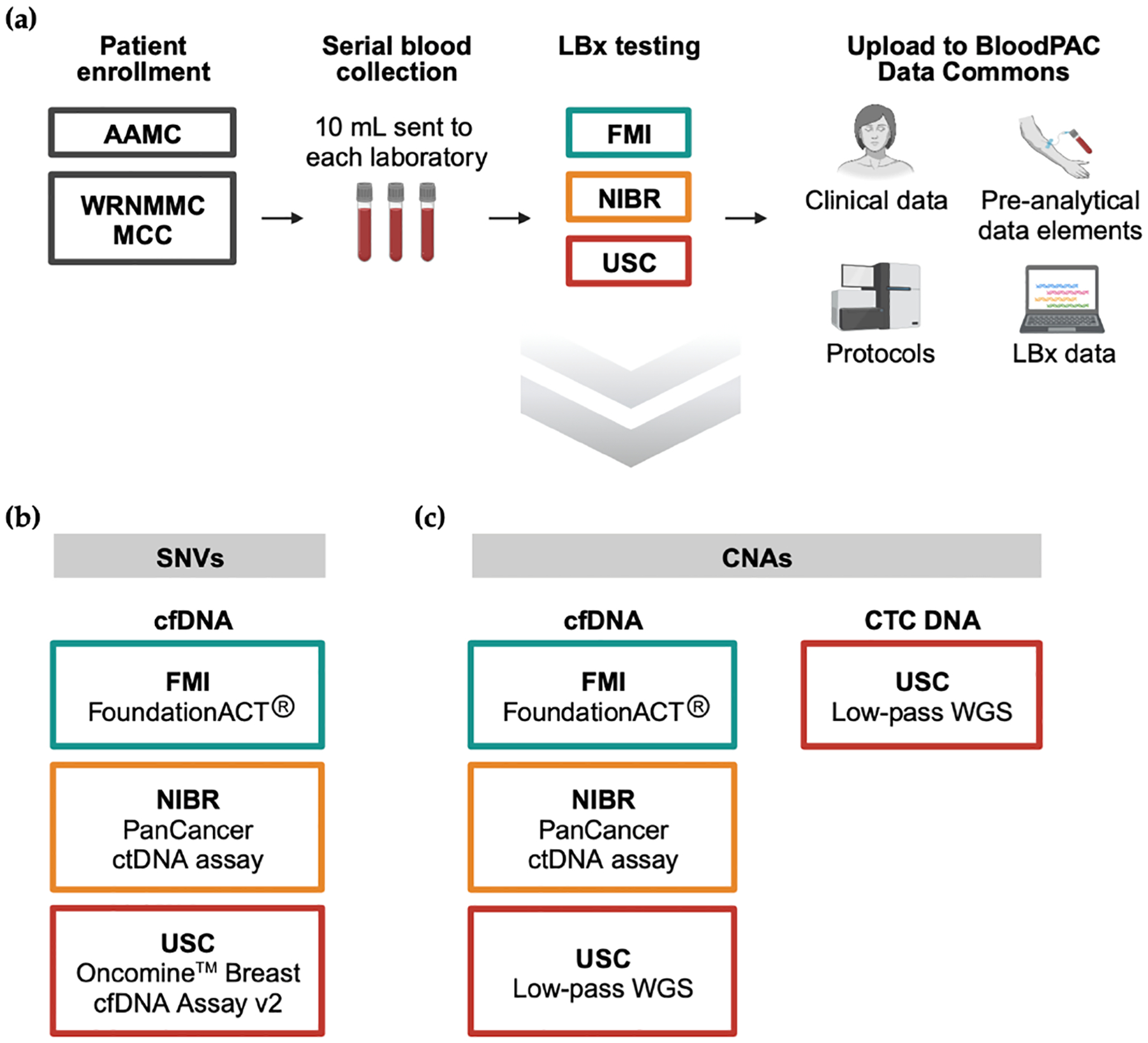
Study design. (**a**) Overview of sample collection, processing, and data aggregation. (**b**,**c**) LBx assays performed by each laboratory for detection of SNVs in cfDNA (**b**) and detection of CNAs in cfDNA and CTC DNA (**c**).

The BloodPAC-007 study enrolled 38 patients from the WRNMMC MCC and AAMC clinical sites and a total of 107 blood samples were collected and analyzed (Table 1, Supplementary Table S2). At each testing site, samples were assayed for cfDNA SNVs and CNAs with FMI using the CLIA-certified FoundationACT assay, NIBR utilizing a laboratory-developed PanCancer ctDNA assay for both SNV and CNA detection, and USC using a combination of the Oncomine Breast cfDNA Assay v2 for SNV detection with a previously published lpWGS method for CNA profiling (Methods, Figure 1). In addition to cfDNA, USC also applied the enrichment-free HDSCA platform to identify CTCs, which were subjected to the same lpWGS method for CNA analysis. Each laboratory carried out the analysis of sequencing data for their own set of samples using the pipeline developed for the assay used. A final list of variants detected by each laboratory together with patient clinical data elements, assay protocols, and preanalytical data elements were subsequently uploaded to the BloodPAC Data Commons.

### 3.2. cfDNA Mutational Profiles via Targeted Sequencing

**Figure 2.**
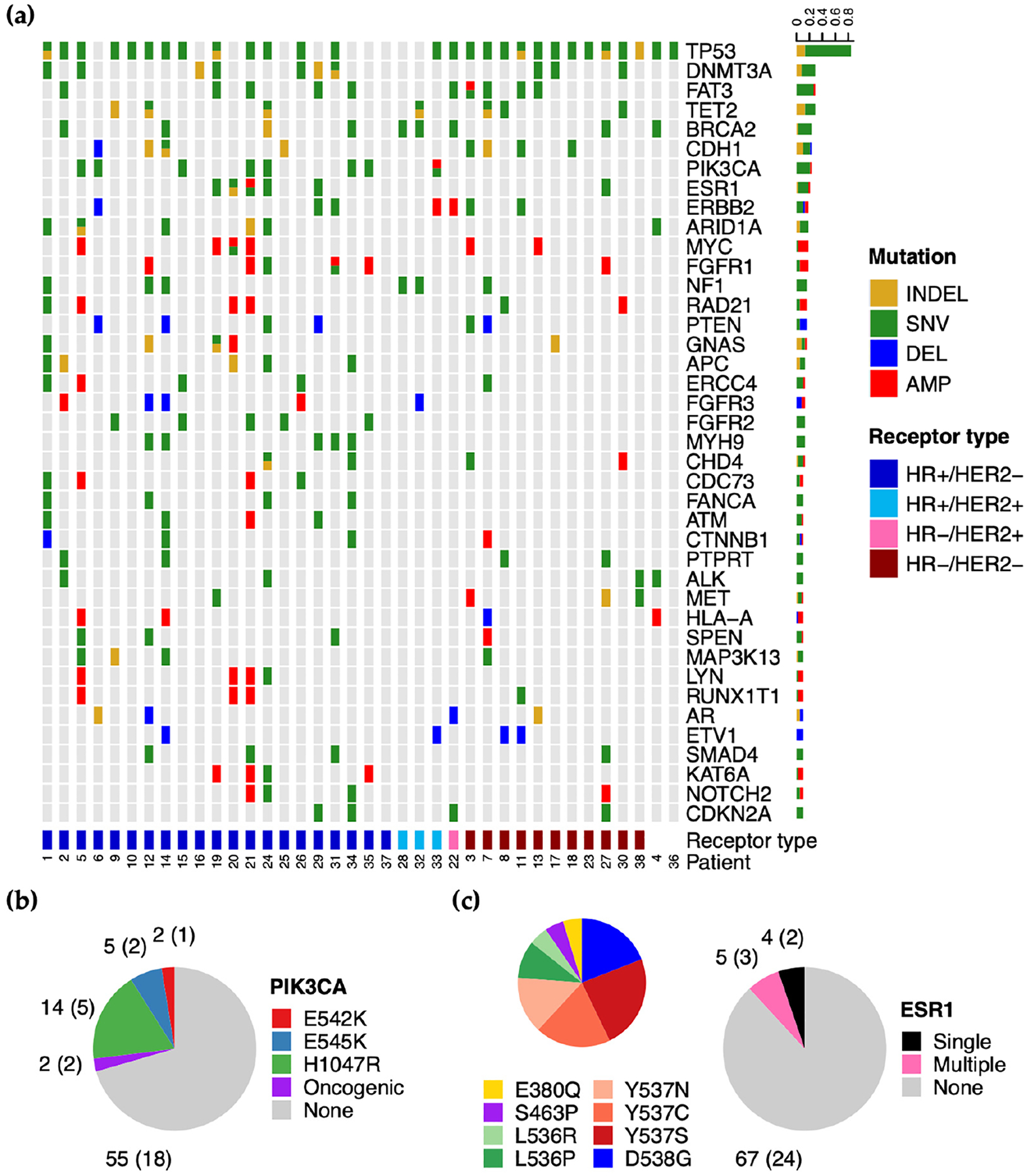
cfDNA genomic variants detected via targeted sequencing. (**a**) SNVs, indels, and CNAs in the 25 most frequently altered COSMIC genes reported by any of the FMI, NIBR, or USC targeted sequencing assays. Plot displays the collective mutation profile of all samples from each patient. Bar graph on the right represents the proportion of patients with each type of mutation for individual genes. Patient ID and receptor type are annotated below the plot. (**b**) Breakdown of clinically actionable PIK3CA mutations detected across samples from HR+ patients. Labels denote the number of samples (patients) with the indicated mutation. (**c**) Similar to (**b**), breakdown of samples with single, multiple, or no clinically actionable ESR1 mutations with additional pie chart showing the prevalence of each variant amongst total variants detected.

Targeted sequencing was performed on 107 cfDNA samples from 38 patients, with 98 samples analyzed by FMI, 96 analyzed by NIBR, and 23 analyzed by USC (Supplementary Table S3). Amongst samples, the most commonly detected mutations in COSMIC genes included SNVs and indels in *TP53*, *FAT3*, *BRCA2*, *CDH1*, *PIK3CA*, *ESR1*, *ARID1A*, and *RB1*, amplification and SNVs in *ERBB2*, and amplification of *MYC* (Figure 2a). Mutations in *DNMT3A*, *TET2*, and *ASXL1* were also detected at high frequency; however, genes have been associated with clonal hematopoiesis (CH) [41] and the analysis methods used in this study were not set up to exclude variants of hematopoietic origin.

Clinically actionable mutations in *PIK3CA* and *ESR1* were detected across 27/76 (35.5%) samples from 10/25 (40%) HR+ patients. Actionable *PIK3CA* mutations were found in twenty-one samples from eight patients, with H1047R being the most common variant, followed by E545K and E542K (Figure 2b). Actionable *ESR1* mutations were found in nine samples from four patients, with five out of nine samples harboring multiple mutations (Figure 2c). Of the total actionable *ESR1* mutations detected, those affecting codon Y537 were most common (Y537N, Y537C, and Y537S), followed by mutations encoding the D538G variant.

### 3.3. Concordance of Cross-Platform cfDNA SNV Detection

**Figure 3.**
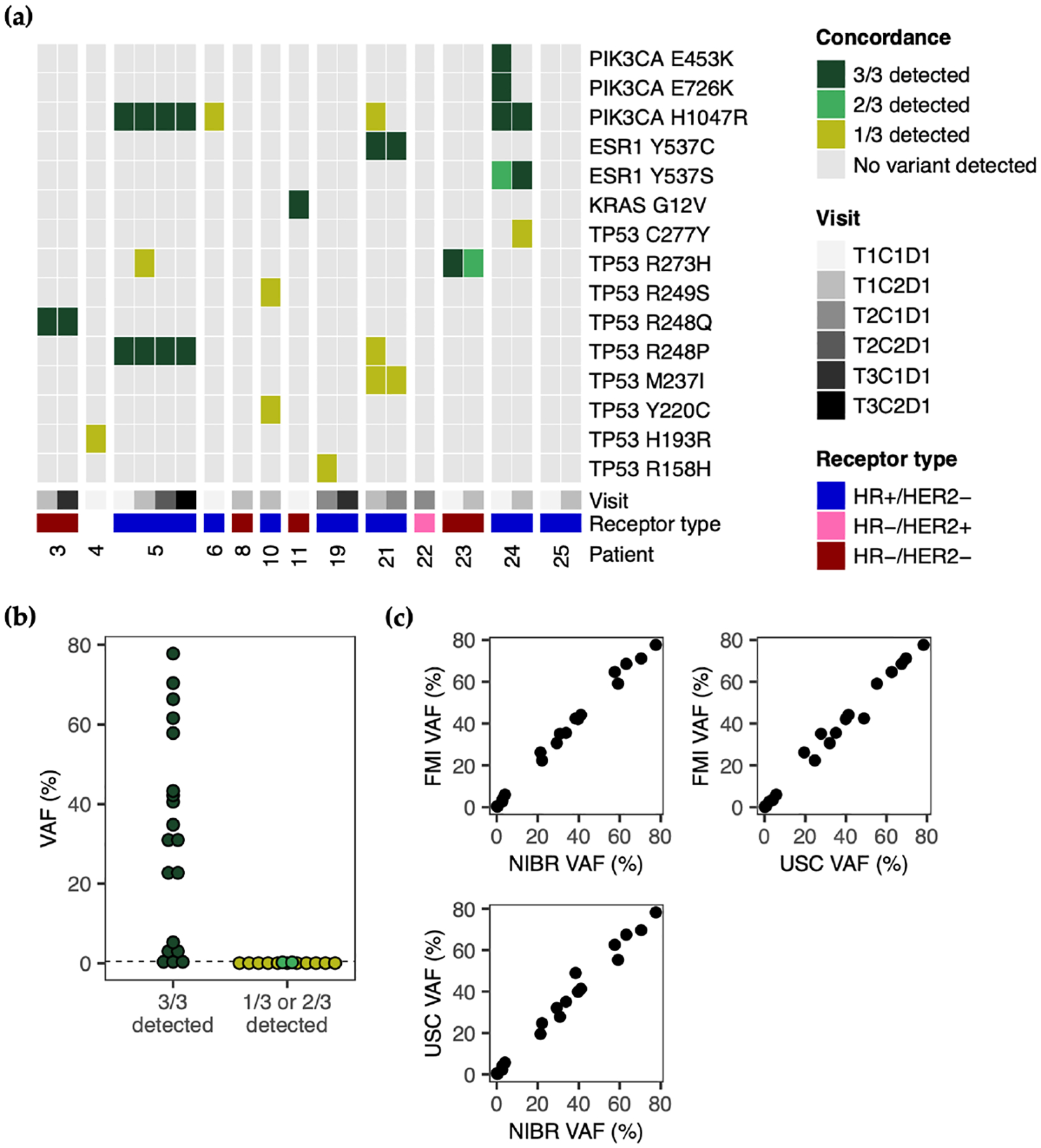
Concordance and clinically actionable mutations. (**a**) Mutations detected in shared target regions for the subset of samples tested by all three laboratories. Variant colors represent detection by 3/3, 2/3, or 1/3 tests (or no variant detected by any test) (**b**) VAFs of variants from (**a**), grouped by inter-assay concordance. Data points are colored according to the legend in (**a**). Dashed line indicates 0.5% VAF. (**c**) Correlation between VAFs reported by each laboratory for pairwise concordant variants.

Inter-assay concordance was assessed for 22 samples from 13 patients, which were analyzed by all three laboratories. In twelve genes with shared target regions across sequencing panels, nineteen (59%) variants were detected by all three platforms, two (6%) were detected by two out of three, and eleven (34%) were detected by one out of three (Figure 3a). All 13 variants detected by only one or two platforms were reported at VAFs below 0.5% (Figure 3b), which is the lowest common limit of detection (LoD) across assays, with only the Oncomine Breast cfDNA Assay v2 having an LoD below this at 0.1% VAF. The 19 three-way concordant variants spanned a wide range of VAFs (0.28–77.80%) and 16/19 were detected above the 0.5% VAF level (Figure 3b). Concordant VAFs reported by each platform were highly correlated with mean absolute differences ranging from 1.94 to 2.18% VAF (USC and FMI: Pearson r = 0.994, mean difference = 1.94%; USC and NIBR: Pearson r = 0.993, mean difference = 2.18% VAF; FMI and NIBR: Pearson r = 0.997, mean difference = 2.04%) (Figure 3c).

### 3.4. cfDNA Whole-Genome CNA Profiles

**Figure 4.**
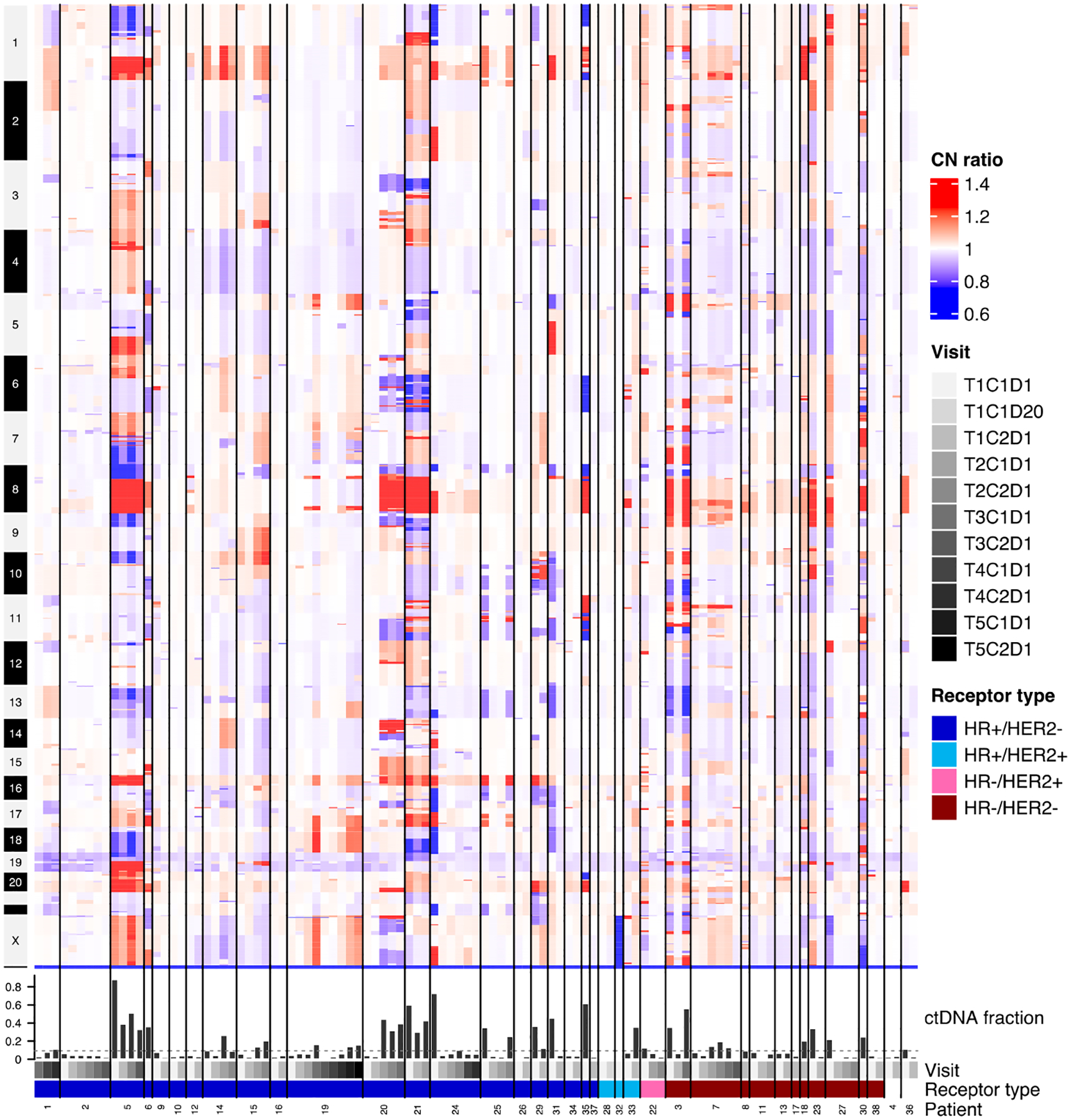
cfDNA whole-genome CNA profiles. Heatmap displays copy number values as ratios to the genome-wide mean across 5000 genomic bins. Regions of copy number gain are shown in red, while regions of copy number loss are shown in blue. Bar plot displays the ctDNA fraction estimated from each profile, where the dashed line indicates the 0.1 threshold. Samples from the same patient are grouped and arranged in chronological order (shown as clinic visits). Patient ID number and receptor type are also annotated below the plot.

In addition to cfDNA mutational profiling via targeted sequencing, copy number profiling via lpWGS was also performed for 105 samples from 38 patients at USC. Longitudinal samples from the same patient generally exhibited similar alteration patterns, while considerable interpatient heterogeneity was observed (Figure 4). Copy number gains on chromosomes 1q and 8q were amongst the few alterations found in multiple patients. The whole-genome copy number profiles were also used to estimate the fraction of ctDNA in each sample. This value varied widely with 38/107 (36%) of samples having a ctDNA fraction > 0.1. For some patients (e.g., 20, 25, and 3), fluctuations in ctDNA fraction from >0.1 to <0.1 were observed across visits (Supplementary Table S4).

### 3.5. CNA Profiling in CTCs and cfDNA

**Figure 5.**
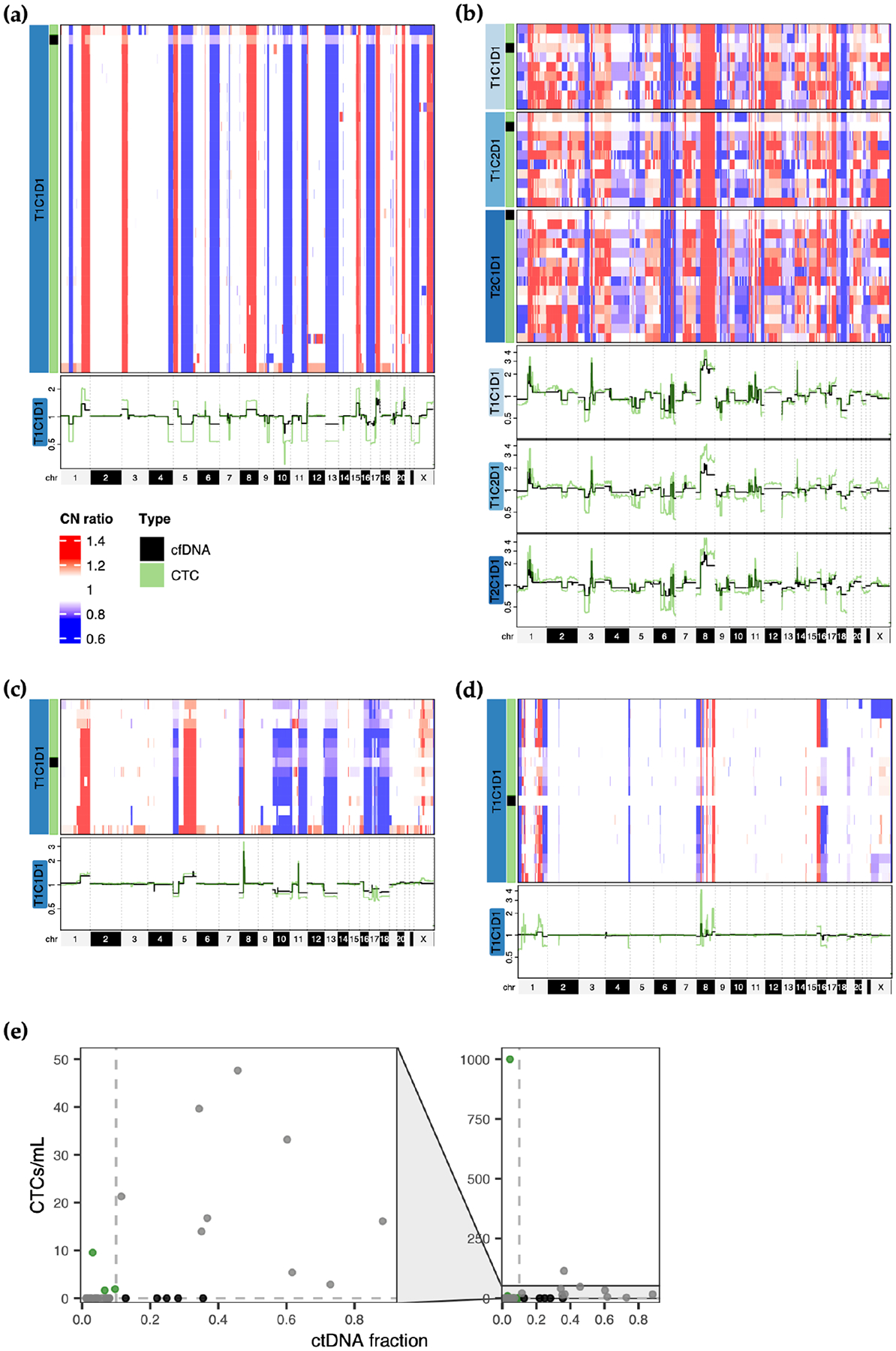
Comparison of CTC and cfDNA CNAs. (**a**–**d**) Whole-genome copy number profiles for CTCs and cfDNA from the same blood tube for patients 6, 21, 31, and 12. Heatmap rows depict profiles for individual CTCs (patient 6: n = 35, patient 21: n = 30, patient 31: n = 13, patient 12: n = 18) or cfDNA and are colored by the ratio to the genome-wide mean according to the scale shown in (**a**). An overlay of the cfDNA (black) and averaged CTC (green) profiles are also shown below each heatmap, along with chromosome numbers. (**e**) Abundance of CTCs (in cells/mL blood) compared to ctDNA fraction at the first study visit for 37 patients. Dashed lines indicate cutoffs for positivity. Green datapoints represent samples with CTCs detected but no ctDNA (fraction < 0.1), while black datapoints represent the opposite case (ctDNA fraction ≥ 0.1 and 0 CTCs).

Matched cfDNA and CTC CNAs were assessed by lpWGS for thirteen samples from eight patients (Figure 5a–d, Supplementary Figure S1a–d). Overall, cfDNA whole-genome profiles closely reflected alterations shared amongst CTCs (Figure 5a–c, Supplementary Figure S1c,d). In two patients with CTCs and cfDNA from multiple timepoints, alterations were also consistently detected across samples (Figure 5b, Supplementary Figure S1d).

We observed cases where CTCs were complementary to cfDNA for CNA profiling. For instance, subclonal CNAs observed in CTCs, such as losses on chr 3p and 9p in patient 31 and losses on chr X in patient 12, were absent in the corresponding cfDNA profiles (Figure 5c,d). Samples from patients 12, 10, and 14 were also cases where CTCs displayed clonal alterations while cfDNA profiles lacked detectable CNAs (Figure 5d, Supplementary Figure S1a,b). Along these lines, when comparing CTCs and ctDNA in 37 samples from the first study visit (cycle 1, day 1 of the initial therapy at enrollment, except for one patient where the first study visit occurred at cycle 2, day 1), 4/37 (11%) samples contained detectable CTCs but not ctDNA, while the opposite was true for 5/37 (14%) samples (Figure 5e).

### 3.6. Longitudinal LBx Profiles

**Figure 6.**
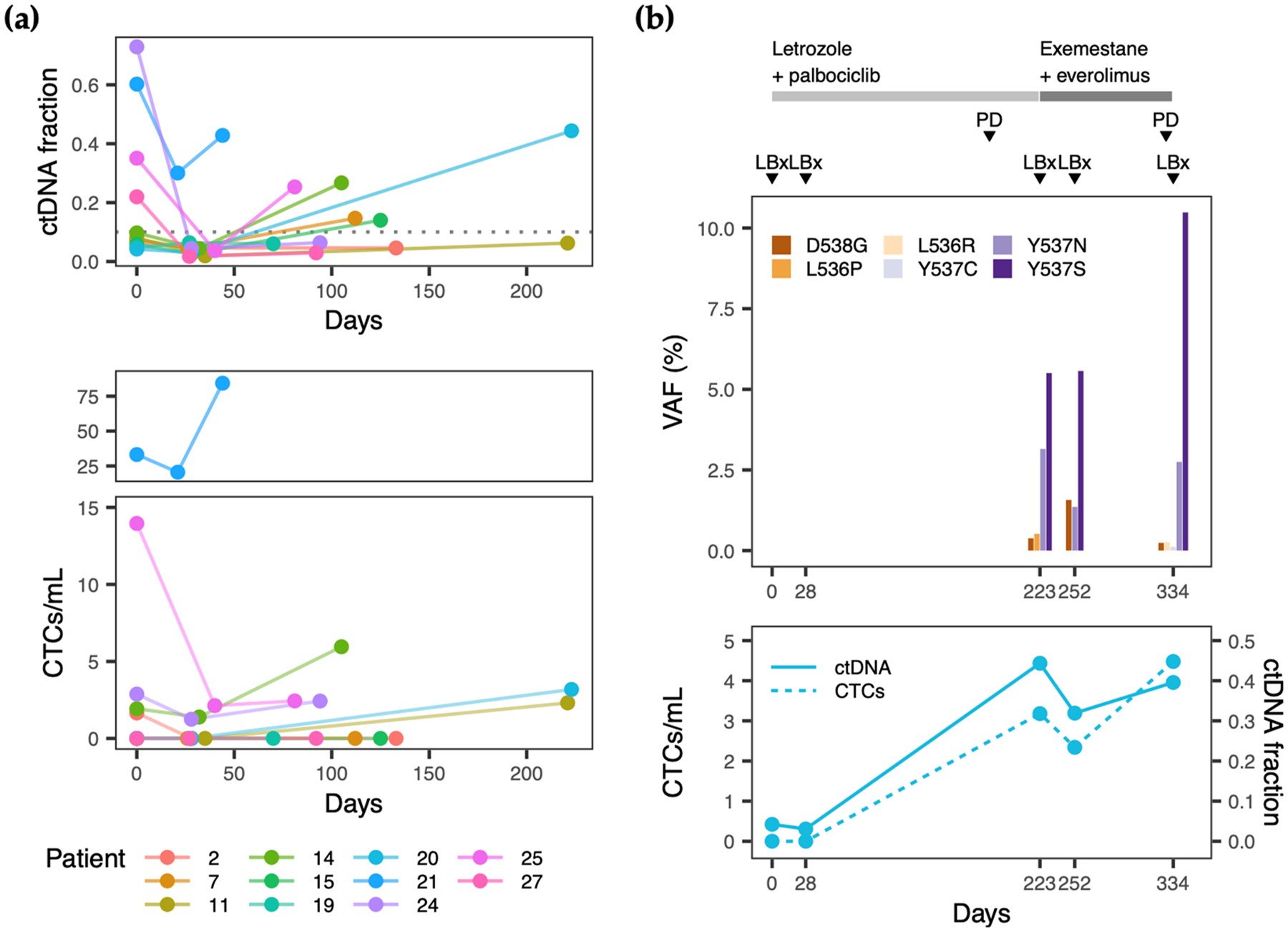
Longitudinal LBx dynamics. (**a**) Levels of ctDNA fraction and CTCs/mL for 11 patients with three consecutive blood draws. (**b**) Longitudinal analysis of five consecutive blood draws from patient 20. Timing of first- and second-line therapies, disease progression, and LBx sample collection are shown at the top. VAFs of ESR1 mutations detected in cfDNA are plotted in the middle section. CTC and ctDNA levels are plotted at the bottom. (PD, progressive disease).

A longitudinal analysis was performed on 11 patients for whom three consecutive blood draws were collected at cycle 1, day 1 (C1D1) and cycle 2, day 1 (C2D1) of the initial therapy at enrollment and at C1D1 of the next line of therapy, typically following a disease progression event (Supplementary Figure S2). The interval between C1D1 of the first therapy and subsequent therapy ranged from 44 to 223 days (Figure 6a, Supplementary Figure S2). Although the absolute levels of ctDNA and CTCs varied across patients and time, relative changes in ctDNA abundance exhibited a consistent pattern with 10/11 of patients showing a decrease between C1D1 and C2D1 and 9/11 patients showing an increase between C2D1 and the progression/C1D1 timepoint of the next line of therapy. CTC levels also followed a similar pattern, although to a lesser extent (5/11 patients showed decreasing levels between C1D1 and C2D1; 6/11 patients showed increasing levels between C2D1 and C1D1 of the next line of therapy).

There was one patient in which LBx results revealed therapy-related changes near the time of disease progression. Patient 20 was an HR+/HER2− patient who was enrolled with de novo metastatic disease and had targeted sequencing of five consecutive cfDNA samples throughout treatment (C1D1 and C2D1 of treatment 1, C1D1 and C2D1 of treatment 2, and C1D1 of treatment 3). Clinically actionable mutations in the *ESR1* ligand-binding domain were detected at C1D1 of treatment 2, occurring 223 days after starting first-line endocrine therapy and in all samples thereafter (Figure 6b). Comparison of LBx results with the clinical timeline further showed the coincidence of *ESR1* mutations, rise in ctDNA fraction, and rise in CTCs in this sample with an initial disease progression event determined via positron emission tomography (PET) imaging 42 days prior (Figure 6b). A second disease progression event was also determined using PET imaging after 105 days of second line therapy (328 days from the first study draw). In samples collected during this period, the levels of *ESR1* mutations, ctDNA fraction, and CTCs were maintained (Figure 6b).

## 4. Discussion

This pilot study by institutions from academia, industry, and the defense health agency is aligned with the goals of the BloodPAC consortium and the Cancer Moonshot Initiative [23]. Execution of the study design demonstrates the feasibility of multicenter collaborations to collect and distribute clinical samples as well as generate and harmonize LBx data from different platforms. Though a small number of prior studies have brought together various groups to conduct non-competitive, cross-platform comparisons [42,43], this is one of few studies performed using clinical specimens [44]. Our ability to obtain multiple blood tubes at each study visit enabled testing on identical samples across laboratories, thereby reducing variability in the starting material and allowing the use of standard protocols for individual tests. Ultimately, the results reflect the collective findings of these platforms in the context of real-world application to heterogeneous MBC patient samples.

It is well accepted that mutational profiling of cfDNA can provide information on variants harbored by a tumor, including clinically actionable variants, and has led to cfDNA testing becoming more prevalent for treatment selection in MBC [9,10]. Our targeted sequencing results were consistent with this notion, with several recurrently altered breast cancer genes [45] commonly mutated across samples and actionable mutations corresponding to FDA-approved therapies detected in 40% of HR+ patients. Although the concordance analysis encompassed a limited number of shared target regions amongst the three sequencing panels, we observed perfect inter-assay agreement for variants detected above the 0.5% VAF level, which was the lowest common LoD across assays. All variants detected by only one or two of the three platforms were reported below this level, which is consistent with results from prior studies showing higher discordance rates in low frequency (<0.5–1% VAF) variants [44,46]. Meanwhile, the close agreement between VAFs reported by each platform for concordant variants has not explicitly been examined in similar studies with patient samples. Together, these results are encouraging as they demonstrate high concordance across a wide range of mutant VAFs found in MBC specimens.

Few groups have performed parallel analyses of CTCs and cfDNA in MBC [47,48], let alone genomic analyses from the same blood tube [6,49,50]. The cases analyzed in this study exemplify the complementary nature of CTC and cfDNA profiling, particularly in terms of overcoming low CTC or ctDNA abundance and capturing subclone heterogeneity. While both CTCs and cfDNA were available in most samples at the first study visit, 24% only contained one analyte, with similar proportions of samples having either CTCs or ctDNA. The relationship between CTCs and ctDNA is potentially interesting given the current understanding of their biological and clinical relevance. CTCs are hypothesized to drive seeding of new metastatic sites, possibly enabled by enhanced capabilities for entering and/or surviving in the circulatory environment [51], and are an established prognostic biomarker in MBC [52]. On the other hand, ctDNA is largely attributed to DNA fragments released by apoptotic tumor cells, theoretically those from the therapy-sensitive tumor population during treatment [53], and is also associated with prognosis and tumor burden [11,47]. Despite different driving factors being associated with the presence of CTCs and ctDNA in the blood, their levels seem to somewhat correlate [11,47] and the significance of discordant cases remains to be elucidated.

The longitudinal analysis included a subset of 11 patients with varying receptor subtypes and therapeutic regimens but identified a consistent pattern of acute decrease between the first two cycles of therapy followed by an increase in ctDNA and CTCs near the time of disease progression. A small number of prior studies have attempted to correlate early changes in CTCs and ctDNA with treatment response, but complexities associated with the dynamics of these species have hampered validation of such an approach [54–56]. Serial blood collection at regular intervals throughout the duration of treatment could better assess changes relevant to therapeutic response and is being adopted by some newer clinical trials [57,58].

The index case illustrates the feasibility of a multianalyte LBx approach for disease monitoring and therapy selection. Increases in actionable *ESR1* mutation VAFs, ctDNA fraction, and CTC abundance all coincided with the timing of the first disease progression and levels of these analytes were also sustained between the start of second-line endocrine therapy and a second disease progression event. The most abundant *ESR1* mutation detected, Y537S, confers constitutive, ligand-independent activation of the ER and has been associated with exposure to aromatase inhibitor (AI) therapy [59–61]. The BOLERO-2 trial, which demonstrated benefit from exemestane plus everolimus compared to exemestane alone, also showed decreased progression-free survival for patients with the Y537S mutation compared to those with wild-type *ESR1* in the combination arm (8.48 vs. 4.17 months) [62]. More recently, *ESR1* mutation status was FDA approved to guide the use of the oral selective ER degrader, elacestrant, following progression on at least one line of endocrine therapy [21,63]. Taken together, the LBx results in this example would suggest the emergence of AI-based therapy resistance and benefit from an alternative second-line regimen. As LBx tests for clinically actionable biomarkers gain approval, optimizing the timing of testing for therapeutic decision making will become increasingly important.

A limitation of this study was the lack of matched WBC sequencing or the use of bioinformatic methodologies to identify CH variants. At study inception, standardized approaches to deal with these variants in cfDNA mutational analyses were not widely established. However, given recent evidence [41,64,65], groups such as the BloodPAC consortium have created formal guidance on handling CH variants [24]. Newer bioinformatic methodologies are also being developed to classify tumor-derived versus WBC-derived variants without the need for WBC sequencing [29,66]. The high prevalence of alterations in *DNMT3A*, *TET2*, and *ASXL1* observed in this study further underscores the importance of accounting for CH variants in cfDNA mutational analyses. We also acknowledge that the use of assays with sequencing panels ranging from 12 to 600 genes limited an extensive concordance assessment and more focused studies for specific variants of interest should consider the appropriate assays and patient population needed to conduct such comparisons. Other limitations of this study include the lack of matched tissue sequencing for comparison with detected plasma variants, which could have aided in resolving discordant calls and allowed exploration of tissue-plasma concordance across longitudinal samples. Lastly, the relatively small and heterogeneous patient population did not enable meaningful associations with clinical outcomes.

In conclusion, this study demonstrates a cooperative framework for conducting multicenter LBx studies on clinical samples. Our findings describe the different types of heterogeneity observed in MBC patients using multianalyte, multiplatform testing and could be leveraged for future trial designs to identify comprehensive LBx biomarkers to guide clinical care. Integration of newer LBx assessments, including cfDNA fragmentomics [67,68] and analyses of cancer-associated Evs [69,70], could also be explored to harness additional disease-related information contained in samples collected from patients with MBC. This evolving landscape of novel approaches to study diverse LBx analytes and biofluids should continue to enable progress towards minimally invasive, multidimensional, and personalized cancer profiling.

## Supplementary Material

Supplementary Material

## Figures and Tables

**Table 1.** Study-level clinicopathological information.

Title 1	AAMC	WRNMMC MCC	Total
Patients	18	20	38
Blood draws	51	56	107
Receptor status *			
HR+/HER2−	10	11	21
HR+/HER2+	0	3	3
HR−/HER2+	0	1	1
HR−/HER2−	7	4	11
Histological subtype			
Ductal	13	18	31
Lobular	3	2	5
Other	2	0	2

* One patient excluded from counts due to DCIS and prior mastectomies. One HR+ patient with HER2 status not available was also excluded. Abbreviations: AAMC, Anne Arundel Medical Center; WRNMMC MCC,Walter Reed National Military Medical Center Murtha Cancer Center; HR, hormone receptor; HER2, human epidermal growth factor receptor 2.

## Data Availability

The data presented in this study are openly available in the BloodPAC Commons at https://data.bloodpac.org/discovery/BPDC000142, reference number BPDC000142.
