# Supplementary material for: Liquid Biopsy Profiling with Multiple Tests in Patients with Metastatic Breast Cancer
Source: J Mol Pathol (Basel). Author manuscript; Available in PMC 2026 Jan 29. (PMC12851560; doi:10.3390/jmp5020013)
Supplement: Supplementary Material [file NIHMS2059476-supplement-Supplementary_Material.pdf]

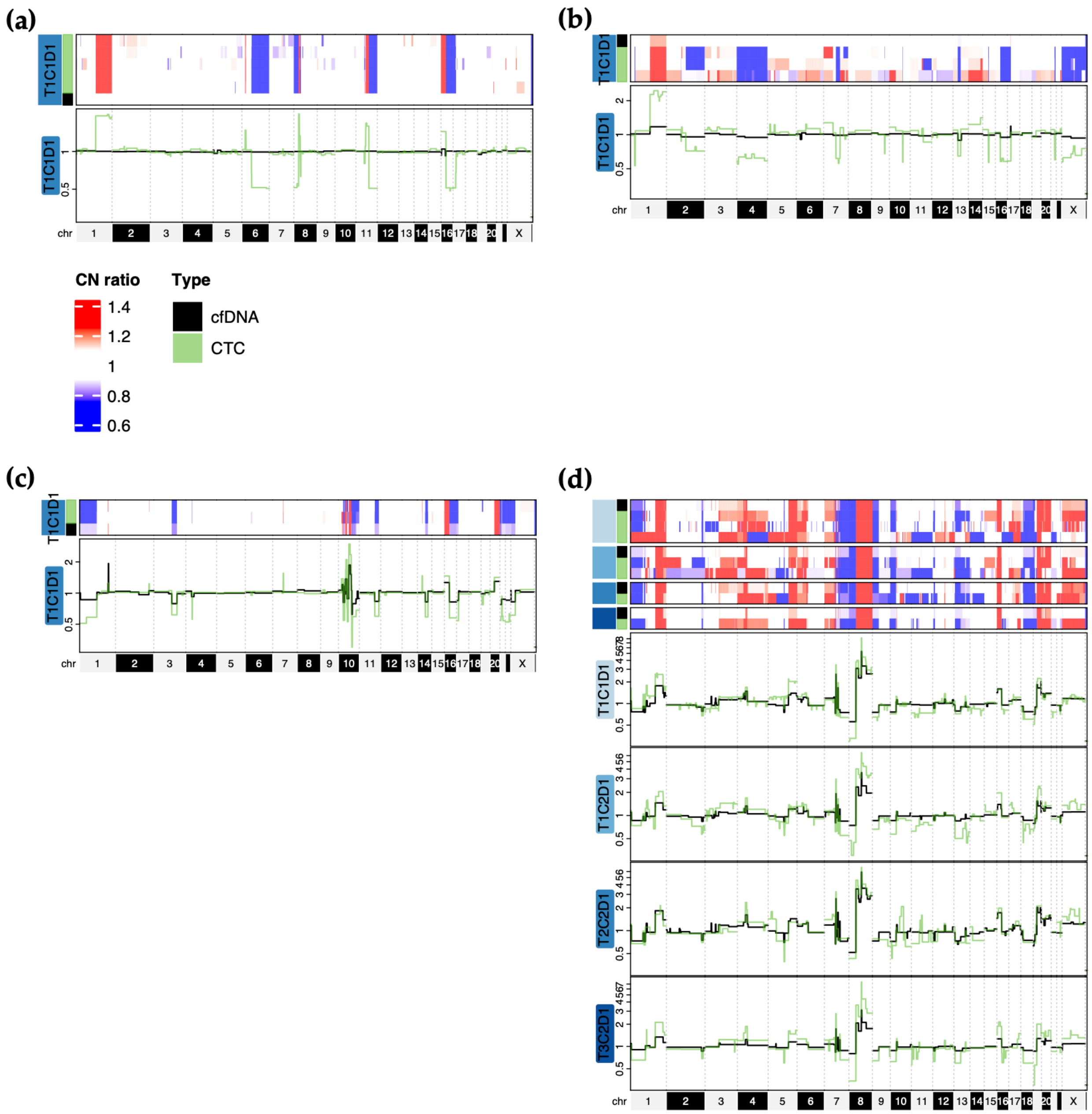

Supplementary Figure S1. Comparison of CTC and cfDNA CNAs for four additional patients. (**a**)-(**d**) Whole-genome copy number profiles for CTCs and cfDNA from the same blood tube for patients 10, 14, 29, and 5. As in Figure 5, heatmap rows depict profiles for individual CTCs (patient 10: n=5, patient 14: n=3, patient 29: n=2, patient 5: n=7) or cfDNA and are colored by the ratio to the genome-wide mean according to the scale shown in (a). An overlay of the cfDNA (black) and single or averaged CTC (green) profiles are also shown below each heatmap, along with chromosome numbers.

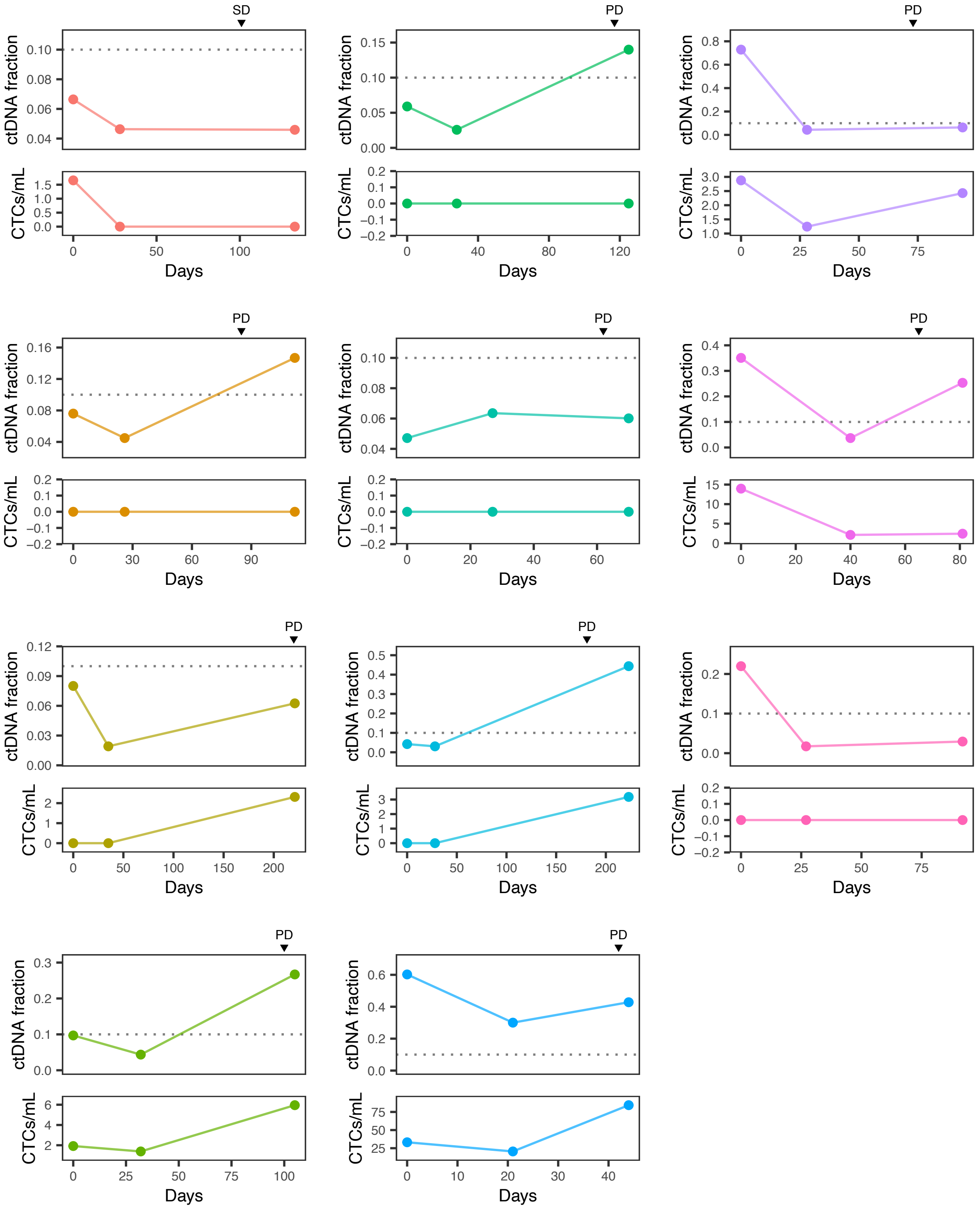

Supplementary Figure S2. LBx dynamics for 11 patients with three consecutive study draws. (Top to bottom, left to right) Patients 2, 7, 11, 14, 15, 19, 20, 21, 24, 25, 27. LBx timepoints correspond to the first day of two cycles of therapy, plus the start of the next line of therapy. Disease status prior to the change in therapy is shown. (PD, progressive disease; SD, stable disease)

Supplementary Table 1

Gene	Alteration(s)	Cancer types	Drugs (for therapeutic implications only)
BRAF	V600E	All Solid Tumors (excluding Colorectal Cancer)	Dabrafenib + Trametinib
ERBB2	Amplification	Breast Cancer	Ado-Trastuzumab Emtansine
ERBB2	Amplification	Breast Cancer	Lapatinib + Capecitabine, Lapatinib + Letrozole
ERBB2	Amplification	Breast Cancer	Margetuximab + Chemotherapy
ERBB2	Amplification	Breast Cancer	Neratinib, Neratinib + Capecitabine
ERBB2	Amplification	Breast Cancer	Trastuzumab + Pertuzumab + Chemotherapy
ERBB2	Amplification	Breast Cancer	Trastuzumab + Tucatinib + Capecitabine
ERBB2	Amplification	Breast Cancer	Trastuzumab Deruxtecan
ERBB2	Amplification	Breast Cancer	Trastuzumab, Trastuzumab + Chemotherapy
ESR1	D538, E380, L469V, L536, S463P, Y537	Breast Cancer	Elacestrant
NTRK1	Fusions	All Solid Tumors	Entrectinib
NTRK1	Fusions	All Solid Tumors	Larotrectinib
NTRK2	Fusions	All Solid Tumors	Entrectinib
NTRK2	Fusions	All Solid Tumors	Larotrectinib
NTRK3	Fusions	All Solid Tumors	Entrectinib
NTRK3	Fusions	All Solid Tumors	Larotrectinib
Other Biomarkers	Microsatellite Instability-High	All Solid Tumors	Pembrolizumab
Other Biomarkers	Tumor Mutational Burden-High	All Solid Tumors	Pembrolizumab
PIK3CA	C420R, E542K, E545A, E545D, E545G, E545K, H1047L, H1047R, H1047Y, Q546E, Q546R	Breast Cancer	Alpelisib + Fulvestrant
PIK3CA	Oncogenic Mutations (excluding C420R, E542K, E545A, E545D, E545G, E545K, Q546E, Q546R, H1047L, H1047R and H1047Y)	Breast Cancer	Alpelisib + Fulvestrant
RET	Fusions	All Solid Tumors (excluding Thyroid Cancer, NSCLC)	Selpercatinib
NTRK1	G595R	All Solid Tumors	Larotrectinib
NTRK3	F617L	All Solid Tumors	Larotrectinib
NTRK3	G623R	All Solid Tumors	Larotrectinib
NTRK3	G696A	All Solid Tumors	Larotrectinib

Supplementary Table 2

Patient	ER	PR	HER2	Histological subtype
1	Positive	Positive	Negative	Ductal
2	Positive	Positive	Negative	Ductal
3	Negative	Negative	Negative	Ductal
*4	no data	no data	no data	Other
5	Positive	Positive	Negative	Ductal
6	Positive	Positive	Negative	Other
7	Negative	Negative	Negative	Ductal
8	Negative	Negative	Negative	Ductal
9	Positive	Positive	Negative	Ductal
10	Positive	Positive	Negative	Lobular
11	Negative	Negative	Negative	Ductal
12	Positive	Positive	Negative	Lobular
13	Negative	Negative	Negative	Ductal
14	Positive	Negative	Negative	Lobular
15	Positive	Negative	Negative	Ductal
16	Positive	no data	Negative	Ductal
17	Negative	Negative	Negative	Ductal
18	Negative	Negative	Negative	Ductal
19	Positive	Positive	Negative	Ductal
20	Positive	Positive	Negative	Ductal
21	Positive	Positive	Negative	Ductal
22	Negative	Negative	Overexpressing	Ductal
23	Negative	Negative	Negative	Ductal
24	Positive	Positive	Negative	Ductal
25	Positive	Positive	Negative	Lobular
26	Negative	Positive	Negative	Ductal
27	Negative	Negative	Negative	Ductal
28	Positive	Positive	Overexpressing	Ductal
29	Positive	Positive	Negative	Ductal
30	Negative	Negative	Negative	Ductal
31	Positive	Positive	Negative	Lobular
32	Positive	Positive	Overexpressing	Ductal
33	Negative	Positive	Overexpressing	Ductal
34	Positive	Positive	Negative	Ductal
35	Positive	Negative	Negative	Ductal
36	Positive	Negative	no data	Ductal
37	Positive	Positive	Negative	Ductal
38	Negative	Negative	Negative	Ductal

*Note: Patient had prior mastectomies for both breasts for ADH (left) and DCIS (right).

Supplementary Table 3

Patient	Number of samples analyzed	Timepoints analyzed	Number of samples analyzyed by the FoundationACT	Number of samples analyzed by the PanCancer ctDNA assay	Number of samples analyzed by the Oncomine Breast cfDNA Assay v2	Number of cfDNA samples analyzed by low-pass WGS
1	4	T3C1D1, T3C2D3, T4C1D1, T4C2D1	4	4	0	3
2	6	T1C1D1, T1C2D1, T2C1D1, T2C2D1, T3C1D1, T3C2D1	6	6	0	6
3	3	T1C1D1, T1C2D1, T3C1D1	3	3	2	3
4	2	T1C1D1, T1C2D1	2	2	1	2
5	4	T1C1D1, T1C2D1,T2C2D1, T3C2D1	4	4	4	4
6	1	T1C1D1	1	1	1	1
7	6	T1C1D1, T1C2D1, T2C1D1, T2C2D1, T3C1D1, T3C2D1	6	6	0	6
8	1	T1C2D1	1	1	1	1
9	3	T1C1D1, T1C2D1, T1C2D2	2	2	0	2
10	2	T1C1D1, T1C2D1	2	2	1	2
11	3	T1C1D1, T1C2D1, T2C1D1	3	2	1	3
12	2	T1C1D1, T1C2D1	2	2	0	2
13	2	T1C1D1, T1C2D1	2	2	0	2
14	4	T1C1D1, T1C2D1, T2C1D1, T2C2D1	4	4	0	4
15	4	T1C1D1, T1C2D1, T2C1D1, T2C2D1	4	2	0	4
16	2	T1C1D1, T1C2D1	0	2	0	2
17	1	T1C2D1	1	1	0	1
18	1	T1C2D1	1	0	0	1
19	9	T1C1D1, T1C2D1, T2C1D1, T3C1D1, T3C2D1, T4C1D1, T4C2D1, T5C1D1, T5C2D1	7	9	2	9
20	5	T1C1D1, T1C2D1, T2C1D1, T2C2D1, T3C1D1	5	5	0	5
21	3	T1C1D1, T1C2D1, T2C1D1	2	3	3	3
22	3	T1C1D1, T2C1D1, T2C2D1	3	3	1	3
23	2	T1C1D1, T1C2D1	2	2	2	2
24	6	T1C1D1, T1C2D1, T2C1D1, T2C2D1, T4C1D1, T4C2D1	6	6	2	6
25	4	T1C1D1, T1C2D1, T2C1D1, T2C2D1	4	4	2	4
26	2	T1C1D1, T1C2D1	1	2	0	2
27	4	T1C1D1, T1C2D1, T2C1D1, T2C2D1	4	3	0	4
28	2	T1C1D1, T1C1D20	1	2	0	2
29	2	T1C1D1, T1C2D1	2	2	0	2
30	1	T1C1D1	1	1	0	1
31	2	T1C1D1, T1C2D1	2	2	0	2
32	1	T1C1D1	1	1	0	1
33	2	T1C1D1, T1C2D1	2	2	0	2
34	2	T1C1D1, T1C2D1	1	2	0	2
35	1	T1C1D1	1	1	0	1
36	2	T1C1D1, T1C2D1	2	0	0	2
37	1	T1C2D1	1	0	0	1
38	2	T1C1D1, T1C2D1	2	0	0	2
Total	107		98	96	23	105

Supplementary Table 4

Patient	Visit	ctDNA fraction
1	T3C1D1	0.0307
1	T4C1D1	0.08197
1	T4C2D1	0.1155
2	T1C1D1	0.06642
2	T1C2D1	0.04633
2	T2C1D1	0.04591
2	T2C2D1	0.04429
2	T3C1D1	0.04318
2	T3C2D1	0.02398
3	T1C1D1	0.3552
3	T1C2D1	0.0661
3	T3C1D1	0.5622
4	T1C1D1	0.02439
4	T1C2D1	0.03647
5	T1C1D1	0.8825
5	T1C2D1	0.3922
5	T2C2D1	0.5114
5	T3C2D1	0.3328
6	T1C1D1	0.3623
7	T1C1D1	0.07599
7	T1C2D1	0.04488
7	T2C1D1	0.1468
7	T2C2D1	0.1973
7	T3C1D1	0.1303
7	T3C2D1	0.01972
8	T1C2D1	0.09495
9	T1C1D1	0.07982
9	T1C2D1	0.02136
10	T1C1D1	0.03118
10	T1C2D1	0.04106
11	T1C1D1	0.08006
11	T1C2D1	0.01914
11	T2C1D1	0.06249
12	T1C1D1	0.04509
12	T1C2D1	0.03131
13	T1C1D1	0.06915
13	T1C2D1	0.07177
14	T1C1D1	0.09698
14	T1C2D1	0.0436
14	T2C1D1	0.2669
14	T2C2D1	0.09102
15	T1C1D1	0.05896
15	T1C2D1	0.02572
15	T2C1D1	0.1399
15	T2C2D1	0.2061
16	T1C1D1	0.02689
16	T1C2D1	0.0425
17	T1C2D1	0.03782
18	T1C2D1	0.204
19	T1C1D1	0.04711
19	T1C2D1	0.06355
19	T2C1D1	0.06012
19	T3C1D1	0.1657
19	T3C2D1	0.01705
19	T4C1D1	0.0282
19	T4C2D1	0.06161
19	T5C1D1	0.1434
19	T5C2D1	0.16
20	T1C1D1	0.0421
20	T1C2D1	0.03058
20	T2C1D1	0.4436
20	T2C2D1	0.3193
20	T3C1D1	0.3957
21	T1C1D1	0.6023
21	T1C2D1	0.3003
21	T2C1D1	0.4281
22	T1C1D1	0.1279
22	T2C1D1	0.06662
22	T2C2D1	0.02715
23	T1C1D1	0.3436
23	T1C2D1	0.03516
24	T1C1D1	0.7289
24	T1C2D1	0.04366
24	T2C1D1	0.06383
24	T2C2D1	0.1022
24	T4C1D1	0.059
24	T4C2D1	0.05816
25	T1C1D1	0.3507
25	T1C2D1	0.03687
25	T2C1D1	0.0263
25	T2C2D1	0.2532
26	T1C1D1	0.01148
26	T1C2D1	0.03016
27	T1C1D1	0.2197
27	T1C2D1	0.01727
27	T2C1D1	0.02936
27	T2C2D1	0.025
28	T1C1D1	0.01558
28	T1C1D20	0
29	T1C1D1	0.3672
29	T1C2D1	0.1222
30	T1C1D1	0.2486
31	T1C1D1	0.4569
31	T1C2D1	0.04514
32	T1C1D1	0.015
33	T1C1D1	0.07134
33	T1C2D1	0.3562
34	T1C1D1	0.04128
34	T1C2D1	0.03844
35	T1C1D1	0.6168
36	T1C1D1	0.1152
36	T1C2D1	0.02865
37	T1C2D1	0.02448
38	T1C1D1	0.04145
38	T1C2D1	0.02197
